# The triage role of cytological DNA methylation in women with non-16/18, specifically genotyping high-risk HPV infection

**DOI:** 10.1038/s41416-025-03005-5

**Published:** 2025-04-10

**Authors:** Haiqi Su, Xitong Jin, Linghua Kong, Yan You, Huanwen Wu, Yuligh Liou, Lei Li

**Affiliations:** 1 Department of Obstetrics and Gynecology, Peking Union Medical College Hospital, Beijing, China; 2 National Clinical Research Center for Obstetric & Gynecologic Diseases, Beijing, China; 3 State Key Laboratory for Complex, Severe and Rare Diseases, Peking Union Medical College Hospital, Beijing, China; 4 Department of Medical Laboratory, Beijing Origin-Poly Bio-Tec Co. Ltd, Beijing, China; 5 Department of Pathology, Peking Union Medical College Hospital, Beijing, China; 6 Clinical Precision Medicine Research Center, the First Affiliated Hospital of Guangdong Pharmaceutical University, Guangzhou, China

**Keywords:** Cancer genomics, Diagnostic markers

## Abstract

**Objectives:**

To evaluate cytological DNA methylation testing methods for risk stratification in women with non-16/18 HPV, focusing on high-risk HPV (hrHPV) genotyping.

**Methods:**

This study compared the triage performance of liquid-based cytology (LBC) testing, hrHPV genotyping, and *PAX1*/*JAM3* gene methylation (CISCER) testing. The absolute risks of cervical intraepithelial neoplasia grade 2 or worse (CIN2+), grade 3 or worse (CIN3+), and colposcopy referral rates were calculated.

**Results:**

The CISCER test showed a CIN3+ risk of 39.1% for positive and 0.9% for negative results. In comparison, LBC ≥ ASCUS and HPV33/35 genotyping had CIN3+ risks of 9.8% and 19.3%, respectively, for positive result. The colposcopy referral rates were 17.4% for CISCER+, 61.9% for LBC ≥ ASCUS, and 8.9% for HPV33/35+ genotyping. The CIN3+ risks were 40.0% and 50.0% when CISCER+ was combined with LBC ≥ ASCUS and HPV33/35+, respectively. The CIN3+ risks were 0.0% and 1.0% when CISCER- was combined with LBC with no intraepithelial lesions or malignancy (NILM) and non-HPV33/35, respectively. Our analysis of CIN2+ patients yielded similar results.

**Conclusions:**

DNA methylation testing outperformed LBC in triaging women with non-16/18 hrHPV infections, significantly reducing unnecessary colposcopy referrals, particularly when combined with HPV33/35 genotyping.

## Introduction

Persistent infection with high-risk human papillomavirus (hrHPV) is a major cause of invasive cervical cancer [1, 2]. The 2019 ASCCP Consensus Guidelines for Risk-Based Management recommend direct colposcopy for the primary screening of HPV-16/18 (+) and cytology for triage for other women with non-16/18 hrHPV (+) [3, 4]. However, cytology reliance on specialised infrastructure, trained personnel, and frequent screenings makes it less suitable for widespread use in resource-limited settings [2, 5]. Managing atypical squamous cells of undetermined significance (ASCUS) is particularly problematic due to its high frequency and poor reproducibility, often resulting in unnecessary colposcopy referrals [6, 7]. The increase in referral rates for colposcopy examination has led to an increased burden in China [8]. Therefore, replacing the subjective element of cytological pathology with highly reproducible molecular tests would be more beneficial for cervical cancer screening. Available options for molecular markers for HPV triage include p16/Ki-67 dual-staining, HPV genotyping, DNA methylation and HPV mRNA, as well as some newer biomarkers [9–11]. Increased methylation levels have been shown to increase with the duration of HPV infection [12] and increasing cervical intraepithelial neoplasia (CIN) grade, reaching the highest level in patients with cervical cancer [12, 13]. Many studies have provided accumulative evidence that DNA methylation testing can be a promising cervical screening method and a triage method for hrHPV-positive women because of its good performance and reproducibility [14–21].

Currently, there is insufficient research and limited reporting on triage strategies for non-16/18 HPV types. In countries with high rates of referral for colposcopy [22], the importance of HPV genotyping in the early detection of preneoplasia has been confirmed in some regions [23]. For example, the use of HPV16/18/31/33/45 genotyping in the triage of women with HPV infection has been considered because the risk of cervical intraepithelial neoplasia grade 3 or worse (CIN3+) varies among individuals with high-risk HPV genotypes [24, 25]. The results of another study indicate that the 3-year cumulative risk of CIN3+ is highest for HPV16, 33, 31, 18, and 35 [26]. Methylation of the host genes *PAX1* and *JAM3* (*PAX1*^*m*^ and *JAM3*^*m*^) has great potential as a triage method for hrHPV-infected individuals in cervical cancer screening [21, 27]. The latest research findings indicate that in women with non-16/18 hrHPV (+)infection, *PAX1*^*m*^*/JAM3*^*m*^ is superior to cytology for detecting CIN3+. Compared with LBC ≥ ASCUS, *PAX1*^*m*^*/JAM3*^*m*^(+) reduced the number of significant referrals to colposcopy without compromising diagnostic sensitivity [28].

In this study, we evaluated the performance of liquid-based cytology indicative of atypical squamous cells of undetermined significance (LBC ≥ ASCUS), *PAX1/JAM3* gene methylation (CISCER^®^) testing, and hrHPV genotyping in women with non-16/18 hrHPV infection on the basis of multicenter prospective cohorts (METHY3 and METHY4). We also summarise the current management strategies and their challenges for this population with the aim of providing a valuable reference for policymakers to tailor further alternative management strategies or strategies complementary to colposcopy.

## Materials and methods

### Participants and study design

The study population consisted of two cohorts. One cohort was from a multicenter study (METHY3), and the other was from a clinical trial (METHY4). These two studies had the same enrolment criteria and study methods. Their ethical approval numbers are JS-2380 and K-S2021211, respectively, and their registration numbers are NCT04646954 (registered on November 26, 2020) and NCT05290428 (registered on March 12, 2022), respectively. From November 2020 to October 2022, eligible women underwent opportunistic cervical cancer screening consisting of hrHPV genotyping tests and LBC. The residual cytological samples were sent for *PAX1* and *JAM3* gene methylation testing. The inclusion criteria were as follows: aged 18 years or older; an intact cervix; infection with a non-16/18 hrHPV genotype (31, 33, 35, 39, 45, 51, 52, 56, 58, 59, 66, and 68); no serious immunodeficiency condition or HIV infection, no history of organ transplantation, and was not undergoing treatment with immunosuppressive drugs; and provided full consent. The woman was excluded if she had a known malignant tumour of the female genital tract, was under treatment for an active malignant tumour at another site, or was pregnant. Each study subject participated in this study voluntarily and signed an informed consent form. All procedures performed in the study involving human participants were in accordance with the ethical standards of the institutional and National Research Committees.

### Sample collection, LBC tests, and hrHPV genotyping tests

All eligible participants underwent gynaecological examinations and collection of cervical cytology for hrHPV assays, cytological pathology and CISCER tests.

HPV testing was conducted using a 21 subtypes detection kit (HybriBio Ltd., Guangzhou, China) in accordance with the manufacturer’s instructions. The process consisted of three main steps: (1) amplification of HPV DNA by PCR, (2) hybridisation of the amplified DNA with type-specific probes using HybriBio’s proprietary flow hybridisation technology, and (3) identification of 21 HPV genotypes by enzyme immunoassay. This study included women who tested positive for 12 non-HPV16/18 hrHPV types: HPV 31, 33, 35, 39, 45, 51, 52, 56, 58, 59, 66, and 68. The LBC and imaging system manual was used for cytology tests. Cytologists used the 2014 Bethesda system [29] to classify LBC results. Cytological diagnoses were classified as follows: no intraepithelial lesions or malignancy (NILM), atypical squamous cells of undetermined significance (ASCUS), low-grade squamous intraepithelial lesion (LSIL), atypical squamous cells cannot exclude high-grade squamous intraepithelial lesion (ASC-H), atypical glandular cells (AGC), high-grade squamous intraepithelial lesion (HSIL), squamous cell carcinoma (SCC) or adenocarcinoma (AC).

### DNA methylation tests

Methylation was detected by means of the ‘Human PAX1 and JAM3 gene methylation detection kit (real-time PCR)’ for cervical cancer [CISCER^®^, Class III medical devices approved by the National Medical Products Administration (No. 20233400253)] with glyceraldehyde-3-phosphate dehydrogenase (*GAPDH*) as an internal control (OriginPoly Bio-Tec Co., Ltd., Beijing, China) with the SLAN-96S automatic medical PCR analysis system (Shanghai Hongshi Med Tech Co., Ltd., Shanghai, China) per the manufacturer’s instructions. The hypermethylation level of the *PAX1* gene and *JAM3* gene was determined by calculating the difference between the two Ct values (ΔCt*PAX1* = Ct*PAX1* − Ct*GAPDH* and ΔCt*JAM3* = Ct*JAM3* - Ct*GAPDH*). According to the manufacturer’s instructions, the CISCER (+) result was defined as ΔCt*PAX1* ≤ 6.6 or ΔCt*JAM3* ≤ 10.0.

According to the clinical trial summary report of the ‘Human PAX1 and JAM3 gene methylation detection kit (real-time PCR)‘ for cervical cancer [CISCER®, Class III medical device approved by the National Medical Products Administration (No. 20233400253)], the sensitivity of CISCER for detecting CIN3+ in non-16/18 hrHPV-positive women is 95.96%, and the specificity is 87.09%. Sample size calculation for this study was performed with the following parameters: H1: Se > Se0, H1: Sp > Sp0, Power = 0.8, Alpha = 0.05, P(prevalence) = 0.1, Se0 (cytology) = 0.8, Se1 (CISCER) = 0.9596, Sp0 (cytology) = 0.8, Sp1 (CISCER) = 0.8709. The calculation result indicates that a minimum of 300 non-16/18 hrHPV-positive women are required for this study, including 30 CIN3+ patients.

### Histology evaluation

All participants underwent examinations by colposcopic impression, and 2–4 point colposcopy-directed biopsies were performed on the lesions or quadrant biopsy of the normal cervix. When the four-quadrant punch biopsy method was indicated, random biopsies were taken at the 2, 4, 8, and 10 o’clock positions or 3, 6, 9, and 12 o’clock positions. The final diagnostic results were classified at normal cervix, CIN1, CIN2, CIN3 or cervical cancer. All pathological results in this study were obtained from biopsy samples. Two clinical pathologists independently reviewed the histological results.

### Statistical analysis

SPSS 27.0 (IBM Corp., Armonk, NY, USA) and R (version 4.1.2, Vienna, Austria) were used for all the statistical analyses. For normally distributed continuous data, the mean (standard deviation) [Mean(SE)] is used for description, and t-test is applied for group comparisons. For non-normally distributed continuous data, the median and interquartile range [M(Q1, Q3)] are used for representation, and the Mann-Whitney U test is employed for group comparisons. Categorical data are described as frequency and composition ratio N(%), and group comparisons are performed using the x^2^ test or Fisher’s exact test. Logistic regression models were used to calculate odds ratios (ORs), and 95% confidence intervals (CIs) were used to analyse the factors related to cervical intraepithelial neoplasia grade 2 or worse (CIN2+) and CIN3+. Receiver operating characteristic (ROC) curves were used to evaluate the AUCs of different detection methods for CIN2+ vs. CIN2+ and CIN3+ vs. CIN3+. The sensitivity, specificity, positive predictive value (PPV), and negative predictive value (NPV) for detecting CIN2+ and CIN3+ were calculated with 95% confidence intervals (CIs). The receiver operating characteristic (ROC) curves for detecting CIN2+ and CIN3+ were compared with that of LBC ≥ ASCUS by means of McNemar’s test. Separate estimates were retrieved for single and combined triage strategies using LBC ≥ ASCUS, HPV 33/35 genotyping, and CISCER.

Specifically, the performance of seven triage strategies was evaluated: (1) LBC ≥ ASCUS, (2) HPV33/35 genotyping, (3) CISCER, (4) LBC ≥ ASCUS combined with HPV33/35 genotyping, (5) LBC ≥ ASCUS combined with CISCER, (6) HPV33/35 genotyping combined with CISCER and (7) LBC ≥ ASCUS and HPV33/35 genotyping combined with CISCER. Sensitivity and specificity were estimated with Wald 95% confidence intervals (95% CI). All data with pathology results were pooled, and the absolute risk of CIN3+ was calculated. The direct referral percentage was calculated as the percentage of positives from each screening strategy, and the number of referrals needed to detect one CIN2+ or one CIN3+ was calculated by dividing the number of screen positives by the number of true positives. All tests were two-sided, and *P* values of <0.05 with 95% CIs indicate statistically significant differences.

## Results

### Characteristics of the participants

**Fig. 1:**
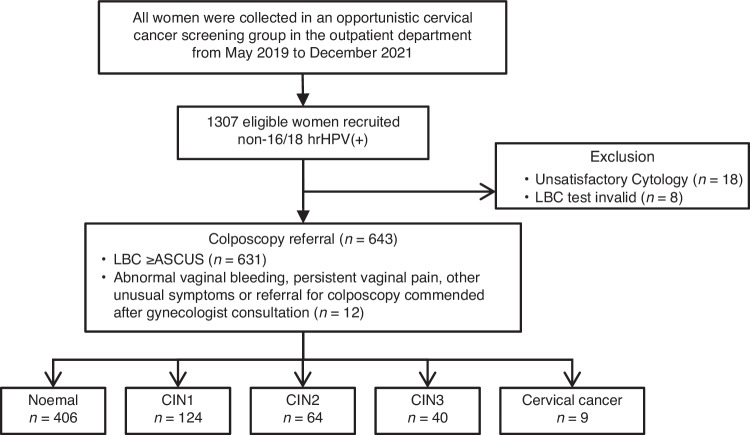
Flowchart of the cervical sample collection cohort. CC cervical cancer, CIN cervical intraepithelial neoplasia, CISCER(+) ΔCt *PAX1* ≤ 6.6 or ΔCt *JAM3* ≤ 10.0, non-16/18 hrHPV(+) infection with HPV31, 33, 35, 39, 45, 51, 52, 56, 58, 59, 66 and/or 68, LBC liquid-based cytology test; NILM negative for intraepithelial lesion or malignancy, ≥ASCUS atypical squamous cells of undetermined significance or worse.

As shown in Fig. 1, a total of 1307 women with non-16/18 hrHPV(+) were included in this study. The characteristics of the participants are summarised in Table 1. In total, 643 women had cervical histology results, and among them, 406 (63.1%) had normal findings or inflammation, 124 (19.3%) had CIN1, 64 (10.0%) had CIN2, 40 (6.2%) had CIN3, and 9 (1.4%) had cervical cancer. The median age was 40 years [interquartile range (IQR): 33–52].

**Table 1:** Clinical characteristics of the participants.

Characteristic	Level	Overall
*n*		643
AGE (median [IQR])		40.00 [33.00, 52.00]
ΔCt *PAX1* (median [IQR])		15.41 [9.31, 17.28]
ΔCt *JAM3* (median [IQR])		15.41 [13.05, 17.07]
Pathology (%)	Normal	406 (63.1)
	CIN1	124 (19.3)
	CIN2	64 (10.0)
	CIN3	40 (6.2)
	CC	9 (1.4)
LBC Results (%)	NILM	245 (38.1)
	ASCUS	208 (32.3)
	LSIL	153 (23.8)
	ASC-H	13 (2.0)
	AGC	3 (0.5)
	HSIL	21 (3.3)
Monotypic or multi severe infections of HPV (%)	1 type	470 (73.1)
	2 types	140 (21.8)
	3 types	24 (3.7)
	4 types	6 (0.9)
	5 types	3 (0.5)
CISCER (%)	(−)	531 (82.6)
	(+)	112 (17.4)

*AGC* atypical glandular cells, ASC-H atypical squamous cells cannot exclude HSIL, *ASCUS* atypical squamous cells of undetermined significance, *CC* cervical cancer, *CIN* cervical intraepithelial neoplasia, *CISCER(+) ΔCt* *PAX1* ≤ 6.6 or ΔCt *JAM3* ≤ 10.0, *HSIL* high-grade squamous intraepithelial lesion, *IQR* interquartile range, *LBC* liquid-based cytology, *LSIL* low-grade squamous intraepithelial lesion, *NILM* no intraepithelial lesions or malignancy.

Among women with definite cervical histology results, 470 (73.1%) were infected with a single HPV genotype, 140 (21.8%) with dual genotypes, and 5.1% with three or more HPV genotypes. The proportion of HPV infections by type is shown in Supplementary Fig. 1.

Among women with definite cervical histology results, 61.9% (398/643) had abnormal cytology results, and among them, 208 (32.3%) had ASCUS, 153 (23.8%) had LSIL, 13 (2.0%) had ASC-H, 3 (0.5%) had AGC, and 21 (3.3%) had HSIL. The positive rate of CISCER was 112/643 (17.4%), the median ΔCt of *PAX1* methylation was 15.41 [IQR: 9.31, 17.28], and the median ΔCt of *JAM3* methylation was 15.41 [IQR: 13.05, 17.07].

### Relationships between LBC results or the *PAX1*^*m*^/*JAM3*^*m*^ tests with cervical pathology

**Fig. 2:**
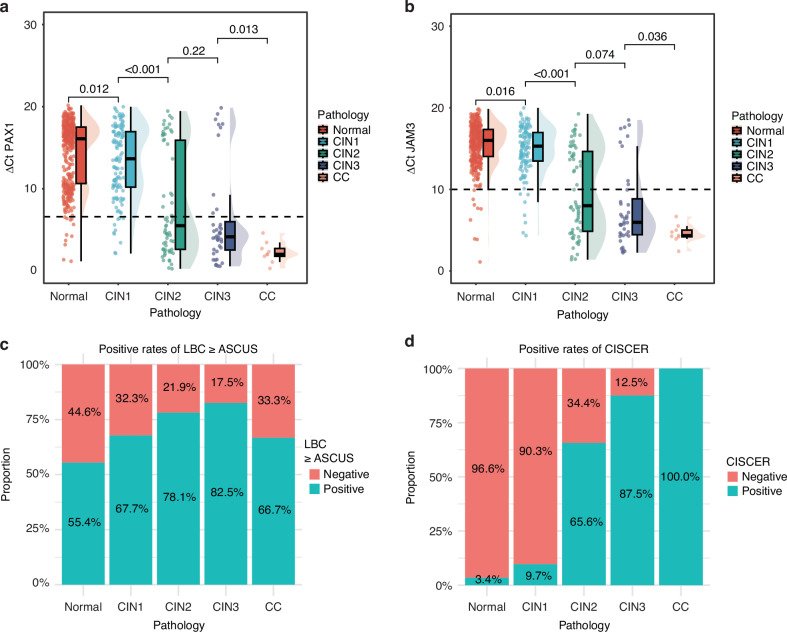
The methylation levels of PAX1 and JAM3 and positive rates of LBC and CISCER are associated with lesion grade. **a** Distribution of the ΔCt of PAX1 methylation on the basis of pathology results; (**b**) distribution of the ΔCt of JAM3 methylation on the basis of pathology results; (**c**) positive rates of LBC ≥ASCUS among different pathological results; (**d**) positive rates of CISCER among different pathological results. CC cervical cancer, CIN cervical intraepithelial neoplasia, CISCER(+) ΔCt *PAX1* ≤ 6.6 or ΔCt *JAM3* ≤ 10.0, LBC liquid-based cytology test, ≥ASCUS atypical squamous cells of undetermined significance or worse.

The gene methylation detection levels among the patients included in the analysis were grouped and compared, with these patients being categorised on the basis of the pathological grading results. The aim of this analysis was to explore the methylation status of genes across diverse lesion states. The median ΔCt values of PAX1 and JAM3 methylation significantly differed between the normal and CIN1 groups, between the CIN1 and CIN2 groups, and between the CIN2 and CIN3 groups (Fig. 2a, b, *P* < 0.05). In the pathology evaluation, the positive rates of LBC ≥ ASCUS were 55.4%, 67.7%, 78.1%, 82.5%, and 66.7% in the normal, CIN1, CIN2, CIN3, and cervical cancer groups, respectively (Fig. 2c), and those of CISCER were 3.4%, 9.7%, 65.6%, 87.5%, and 100.0% in the normal, CIN1, CIN2, CIN3, and cervical cancer groups, respectively (Fig. 2d).

### Logistic regression model analysis

To confirm the risk factors associated with CIN2+ or CIN3+, LBC ≥ ASCUS, CISCER(+) and HPV genotyping (HPV31, HPV33, HPV35, HPV39, HPV45, HPV51, HPV52, HPV56, HPV58, HPV59, HPV66, and HPV68) were incorporated into the multivariate logistic regression model. As shown in Table 2, CISCER(+), LBC ≥ ASCUS, HPV33(+) and HPV35(+) were risk factors for the development of CIN2+. The OR values (95% CI) were 2.43 (1.23–4.81), 51.04 (27.15–95.94), 3.45 (1.21–9.82), and 5.97 (1.36–26.25), respectively. The logistic regression model analysis revealed that only CISCER(+) status was an independent risk factor for CIN3+ status, with an OR of 68.07 (26.09–177.57).

**Table 2:** Multivariate logistic regression analysis of test results related to CIN2+ and CIN3+.

	Total (*N* = 643)	*n* for CIN2+	OR(95%CI)	*P* value	*n* for CIN3+	OR (95%CI)	*P* value
LBC≥ASCUS	398	89	2.43 (1.23–4.81)	**0.01**	39	2.55 (1.25–5.21)	0.318
CISCER(+)	112	86	51.04 (27.15–95.94)	**<0.001**	44	68.07 (26.09–177.57)	**<0.001**
HPV31(+)	47	12	1.56 (0.53–4.59)	0.419	5	1.49 (0.56–3.97)	0.945
HPV33(+)	41	20	3.45 (1.21–9.82)	**0.02**	10	4.66 (2.13–10.19)	0.568
HPV35(+)	17	6	5.97 (1.36–26.25)	**0.018**	2	1.64 (0.36–7.40)	0.981
HPV39(+)	53	4	0.78 (0.2–3.08)	0.722	1	0.22 (0.03–1.61)	0.569
HPV45(+)	9	4	2.71 (0.37–19.84)	0.326	2	3.57 (0.72–17.66)	0.64
HPV51(+)	78	8	0.84 (0.29–2.44)	0.747	3	0.45 (0.14–1.49)	0.681
HPV52(+)	199	38	1.31 (0.64–2.69)	0.463	17	1.20 (0.65–2.22)	0.978
HPV56(+)	77	10	1.35 (0.5–3.67)	0.559	3	0.46 (0.14–1.51)	0.8
HPV58(+)	140	30	1.7 (0.78–3.7)	0.18	14	1.49 (0.78–2.85)	0.632
HPV59(+)	42	4	1.08 (0.3–3.97)	0.903	2	0.59 (0.14–2.51)	0.654
HPV66(+)	63	2	0.29 (0.06–1.49)	0.14	0	0.00 (0.00–Inf)	0.99
HPV68(+)	41	2	0.21 (0.03–1.27)	0.088	0	0.00 (0.00–Inf)	0.991

Bold indicates *P* < 0.05.

*95% CI* 95% confidence interval, *CISCER(+)* Δ*Ct* *PAX1* ≤ 6.6 or ΔCt J*AM3* ≤ 10.0, *LBC* *≥* *ASCUS* Liquid-based cytology indicative of atypical squamous cells of undetermined significance or worse; *OR* odds ratio.

### Clinical performance of triage tools for women with non-16/18 hrHPV infection

**Fig. 3:**
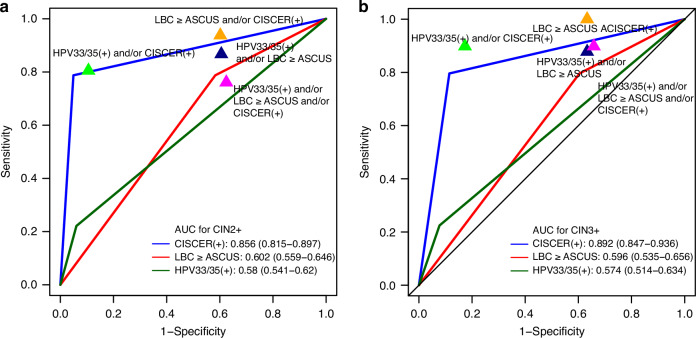
ROC curves of various methods for distinguishing CIN2+ and CIN3+. **a** CIN2+ and **b** CIN3+.

The ROC curves of LBC ≥ ASCUS, CISCER(+), and HPV33/35(+) for CIN2+ and CIN3+ are shown in Fig. 3. The diagnostic accuracy, sensitivity, specificity, PPV, and NPV for triage methods and their combinations for CIN2+ and CIN3+ in the whole cohort are shown in Supplementary Table S1.

There was no significant difference in the AUC for detecting CIN2+ between LBC ≥ ASCUS and HPV33/35(+). However, the AUC for detecting CIN2+ with CISCER(+) was significantly superior to that of LBC ≥ ASCUS (*Z* = 8.63, *p* < 0.001). The AUC of CISCER(+) (0.856 (95% CI: 0.815–0.897)) was higher than that LBC ≥ ASCUS (0.602 (95% CI: 0.559–0.646)). CISCER alone had a sensitivity of 76.1% (95% CI: 68.2-84.0%) and a specificity of 95.1% (95% CI: 93.3–96.9%) for detecting CIN2+. The combined triage strategy of HPV33/35(+) and/or LBC ≥ ASCUS and/or CISCER(+) had the highest sensitivity of 96.5% (95% CI: 93.1–99.9%) but low specificity for CIN2+ (37.5% (95% CI: 33.4–41.7%)).

For detecting CIN3+, CISCER(+) had an AUC of 0.892 (95% CI: 0.847–0.936), which was much higher than those for detecting LBC ≥ ASCUS (AUC 0.596, 95% CI: 0.535–0.656) and HPV33/35(+) (0.574, 95% CI: 0.514–0.634).

### Absolute CIN3+ risks for single and combined triage tests

Supplementary Table S2 shows the absolute CIN2+ risk and absolute CIN3+ risk after the triage of women with non-16/18 hrHPV-infection by LBC ≥ ASCUS, CISCER(+), HPV33/35 genotyping or combinations thereof and referrals needed to detect one CIN3+ with these triage strategies.

**Fig. 4:**
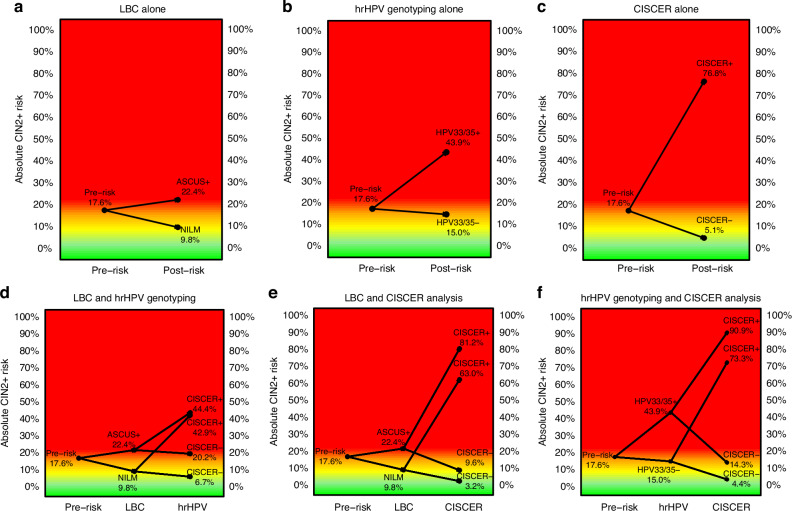
Pre- and post-test CIN2+ risk after applying different triage strategies. Pre- and post-test CIN2+ risk for different diagnostic methods: **a** LBC alone, **b** hrHPV genotyping alone, **c** CISCER alone, **d** LBC and hrHPV genotyping, **e** LBC and CISCER, and **f** hrHPV genotyping and CISCER. ASCUS+ classified as atypical squamous cells of undetermined significance or worse; CISCER*−* ΔCt *PAX1* > 6.6 and ΔCt *JAM3* > 10.0, CISCER+ ΔCt *PAX1* ≤ 6.6 or ΔCt *JAM3* ≤ 10.0; HPV33/35+ HPV33(+) or HPV35(+), HPV33/35- with at least one type of infection among HPV31, 39, 45, 51, 52, 56, 58, 59, 66, and 68; NILM no intraepithelial lesions or malignancy; OR odds ratio. Colours: green, low CIN2+ risk; orange, intermediate CIN2+ risk, red high CIN2+ risk.

**Fig. 5:**
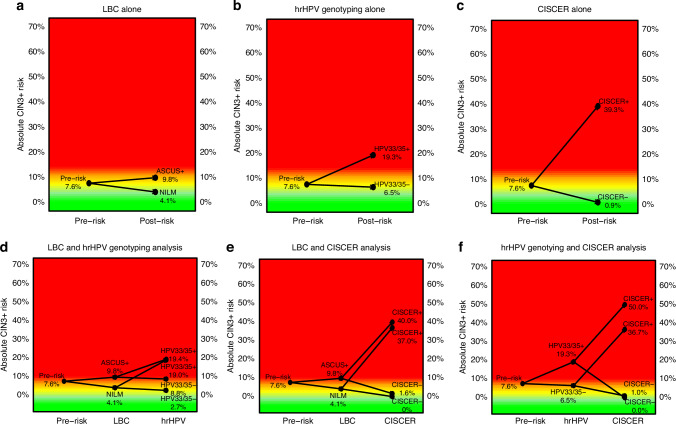
Pre- and post-test CIN3+ risk after applying different triage strategies. Pre- and post-test CIN3+ risk for different diagnostic methods: **a** LBC alone, **b** hrHPV genotyping alone, **c** CISCER alone, **d** LBC and hrHPV genotyping, **e** LBC and CISCER, and **f** hrHPV genotyping and CISCER. ASCUS+ classified as atypical squamous cells of undetermined significance or worse; CISCER− ΔCt *PAX1* > 6.6 and ΔCt *JAM3* > 10.0; CISCER+ ΔCt *PAX1* ≤ 6.6 or ΔCt *JAM3* ≤ 10.0; HPV33/35+ HPV33(+) or HPV35(+); HPV33/35− with at least one type of infection among HPV31, 39, 45, 51, 52, 56, 58, 59, 66, and 68; NILM no intraepithelial lesions or malignancy; OR odds ratio. Colours green, low CIN3+ risk; orange, intermediate CIN3+ risk; red high CIN3+ risk.

Figures 4 and 5 illustrate the pre- and posttest CIN2+ and CIN3+ risks after the application of the single or combined triage strategies, respectively. A positive CISCER test resulted in the highest absolute CIN3+ risk (39.3%, 95% CI: 30.2–49.0%). This strategy resulted in the lowest number of referrals needed to detect one CIN3+ case, and on average, one per 2.5 patients referred was diagnosed with CIN3+ (Supplementary Table S2). Among all the single strategies negativity by CISCER testing yielded an extremely low absolute CIN3+ risk (0.9%, 0.3–2.2%), which was much lower than that of either NILM (4.1%, 2.0–7.4%) or HPV33/35 genotyping negative (6.5%, 4.6–8.8%). The differences in risk between CISCER(-) and CISCER(+), LBC ≥ ASCUS and LBC-NILM, and HPV33/35(+) and HPV33/35(-) were 38.4%, 5.7% and 12.8%, respectively.

The combination of LBC-NILM and CISCER(-) lowered the CIN3+ risk by 0.0% (0.0–1.7%). The highest CIN3+ risk was 50.0% (28.2–71.8%) among women who were HPV33/35(+) and CISCER(+). Compared with women with single CISCER(+), those with LBC ≥ ASCUS and CISCER(+) had the same number of referrals needed to detect one CIN3+, and one per 2.5 patients referred was diagnosed with CIN3+ (Supplementary Table S2).

## Discussion

In this cross-sectional study, we evaluated a strategy based on HPV33/35+ genotyping and CISCER testing for triage non-16/18 HPV-positive women. Our findings demonstrate that CISCER testing showed good discriminative ability, with a CIN3+ risk of 39.1% following positive results and only 0.9% after negative results. Both HPV33/35+ genotyping and CISCER testing could serve as alternative triage methods to cytology, particularly in resource-limited settings. These molecular testing approaches can effectively address the limitations of cytology, which relies heavily on specialised infrastructure, well-equipped laboratories, and highly trained personnel for accurate interpretation.

Previous studies have shown that certain genotypes of high-risk HPV are significantly linked to high-grade cervical lesions (≥CIN2) [30]. In this study, we identified a significant association between the HPV genotypes HPV33 and HPV35 and cervical high-grade lesions by developing a logistic regression model. A recent study from China demonstrated that compared to the current HPV16/18 genotyping combined with cytological triage, triage using HPV16/18/58/33/31 genotyping achieved a lower positivity rate (6.85% vs. 7.35%, *p* = 0.001) while maintaining similar sensitivity (91.35% vs. 96.42%, *p* = 0.32) and specificity (94.09% vs. 93.67%, *p* = 0.56) for the detection of CIN3+ [5]. A prospective multicenter study was conducted to assess the prevalence of HPV types in Asian women diagnosed with invasive cervical cancer. These findings indicated a strong association between HPV16, 18, 52, 45, 58, 33, or 31 and cervical cancer. Notably, the prevalence of HPV33 was found to be higher in Korean women with cervical cancer than in women from other Southeast Asian regions [31]. A comprehensive examination and statistical analysis of the overall risk of CIN in women with normal cytology but positive HPV detection results revealed that the 3-year cumulative risk of CIN3+ was slightly lower for HPV31 than for HPV16 or HPV33. The next most common HPV genotypes were HPV18 and HPV35 [26]. These results were similar to those of our study. Specifically, our investigation revealed a significant difference in the odds ratio for ≥CIN2 associated with the hrHPV genotypes HPV33 or HPV35 infection. Although the current guidelines do not reflect this difference in risk, more aggressive actions, such as direct colposcopy, rather than follow-up after 12 months, might be needed for the higher-risk HPV33/35 genotypes. Large-scale clinical trials or cohort studies are necessary, especially in China, where the prevalence of cervical cancer is high and where HPV genotyping has been widely used in routine screening.

Results in the current literature indicate that CIN2/3 lesions at the highest risk of progression to cervical cancer can be identified by methylation analysis [32]. The results of a recent Finnish study suggested that methylation analysis can serve as a prognostic biomarker to differentiate between regressive and progressive CIN2 lesions [33]. Similar findings can be seen in our study, where CISCER(+) resulted in the highest CIN3+ risk of 39.3% and the largest risk difference between test-positive and test-negative patients (38.4%). Another previous study on methylation indicated that among women who are positive for HPV, the risk of developing CIN2+ is low for those who test negative for methylation [34]. Furthermore, the results of a prospective cohort of women with CIN2/3 with 2 years of follow-up revealed that a negative methylation test is associated with high spontaneous regression [35]. The negative results for CISCER in this study indicate a very low risk of CIN3+ (0.9% (0.3–2.2%)). Collectively, these data suggest that CIN2/3 lesions that are negative for methylation have high regression rates and low risks of cervical cancer progression. Consequently, the analysis of methylation may be considered when determining whether women can return to routine screening.

As a single triage strategy, HPV33/35(+) resulted in an absolute CIN2+ risk of 43.9%, which was much higher than the 22.4% for LBC ≥ ASCUS. HPV33/35(+) resulted in an absolute CIN3+ risk of 19.3%, and the risk difference between test-positive and test-negative patients was 12.8%. In contrast, the absolute risk of LBC ≥ ASCUS resulting in CIN3+ was only 9.8%, and the difference in risk between test-positive and test-negative patients was 5.7%. These findings suggest that risk management in non-16/18 hrHPV-positive women on the basis of HPV33/35 infection status may be more effective than cytology alone. Dun et al. [5] identified that genotyping for HPV16/18/58/33/31 genotyping tests to triage HPV-positive women is of promising use. Another recent meta-analysis examined the cumulative risk of CIN3+ in patients with different HPV genotypes [26]. For non-16/18 hrHPV-positive patients, the risks were 1.5%, 2.5%, and 3.4% at 1, 3, and 5 years, respectively. HPV33 and HPV35 showed higher risks than other non-16/18 types, HPV33 had cumulative risks of 2.7%, 6.1%, and 9.3%, while HPV35 showed risks of 2.2%, 4.4%, and 6.6% at the same time points [26]. From this study, we cannot calculate the cumulative risk at 1, 3, and 5 years, but the statistics show that the immediate risk of CIN3+ in HPV33/35(+) cases is 19.3%, which is higher than the risk of LBC ≥ ASCUS, which is 9.8%. Clinical trials have demonstrated that HPV tests are more sensitive and have a higher long-term negative predictive value than cytology does [36, 37]. On the basis of these findings, we propose a new triage method for determining whether a colposcopy referral is needed for patients with non-16/18 hrHPV infection when cytological test conditions are insufficient.

This study also has several limitations. First, the sample size was relatively small, and all participants were recruited from hospital gynaecological clinics, which may not adequately represent the general population. In particular, regarding HPV genotyping, the risk ratio for HPV33/35 was derived solely from the data obtained in this study, and its reliability needs validation in a larger sample to confirm its true association with the risk of CIN3+. Second, owing to the lack of patient follow-up, we were unable to distinguish the short-term or long-term progression risk of HPV33/35+ and CISCER+. For the long-term cumulative risk of CIN3+ over 1, 3, and 5 years, larger sample sizes and sufficient follow-up data are needed to support and validate the conclusions drawn from this study.

## Conclusions

In conclusion, CISCER(+) is a strong independent risk factor for CIN3+, and its diagnostic accuracy is superior to those of LBC ≥ ASCUS and HPV33/35 genotyping alone. Specifically, the combination of these triage methods can significantly enhance the detection of CIN3+ while minimising unnecessary colposcopy referrals.

## Supplementary information

Table S1. The detection performance of different triage methods for CIN2+ and CIN3+ detection

Table S2. Absolute CIN2+ and CIN3+ risks for single and combined triage tests.

Figure S1. Proportion of infections caused by different HPV genotypes

## Data Availability

All data from this study have been contained in the supplement file.
