# Supplementary material for: The triage role of cytological DNA methylation in women with non-16/18, specifically genotyping high-risk HPV infection
Source: Br J Cancer. 2025 Apr 10;132(11):1064–71. doi: 10.1038/s41416-025-03005-5 (PMC12119919; doi:10.1038/s41416-025-03005-5)
Supplement: Supplementary file 1 — Table S1. The detection performance of different triage methods for CIN2+ and CIN3+ detection [file 41416_2025_3005_MOESM1_ESM.docx]

**Supplement Table S1.** **The detection performance of different triage methods for CIN2+ and CIN3+ detection**

|  | AUC (95% CI) | Sensitivity, % (95% CI) | Specificity, % (95% CI) | PPV, % (95% CI) | NPV, % (95% CI) | P value |
| --- | --- | --- | --- | --- | --- | --- |
| The detection performance of different triage methods for CIN2+ | | | | | | |
| LBC≥ASCUS | 0.602 (0.559-0.646) | 78.8 (71.2-86.3) | 41.7 (37.5-45.9) | 22.4 (18.3-26.5) | 90.2 (86.5-93.9) |  |
| CISCER(+) | 0.856 (0.815-0.897) | 76.1 (68.2-84) | 95.1 (93.3-96.9) | 76.8 (69-84.6) | 94.9 (93-96.8) | <0.001 |
| HPV33/35(+) | 0.58 (0.541-0.62) | 22.1 (14.5-29.8) | 94 (91.9-96) | 43.9 (31-56.7) | 85 (82.1-87.9) | 0.498 |
| LBC≥ASCUS and/or CISCER(+) | 0.668 (0.638-0.699) | 93.8 (89.4-98.2) | 39.8 (35.6-44) | 24.9 (20.8-29.1) | 96.8 (94.4-99.1) | <0.001 |
| HPV33/35(+) and/or LBC≥ASCUS | 0.631 (0.593-0.668) | 86.7 (80.5-93) | 39.4 (35.3-43.6) | 23.4 (19.3-27.4) | 93.3 (90-96.6) | 0.031 |
| HPV33/35(+) and/or CISCER(+) | 0.85 (0.811-0.889) | 80.5 (73.2-87.8) | 89.4 (86.8-92.1) | 61.9 (54.1-69.8) | 95.6 (93.8-97.4) | <0.001 |
| HPV33/35(+) and/or LBC≥ASCUS and/or CISCER(+) | 0.67 (0.643-0.697) | 96.5 (93.1-99.9) | 37.5 (33.4-41.7) | 24.8 (20.7-28.8) | 98 (96.1-99.9) | <0.001 |
| The detection performance of different triage methods for CIN3+ | | | | | | |
| LBC≥ASCUS | 0.596 (0.535-0.656) | 79.6 (68.3-90.9) | 39.6 (35.6-43.5) | 9.8 (6.9-12.7) | 95.9 (93.4-98.4) |  |
| CISCER(+) | 0.892 (0.847-0.936) | 89.8 (81.3-98.3) | 88.6 (86-91.1) | 39.3 (30.2-48.3) | 99.1 (98.2-99.9) | <0.001 |
| HPV33/35(+) | 0.574 (0.514-0.634) | 22.4 (10.8-34.1) | 92.3 (90.1-94.4) | 19.3 (9.1-29.5) | 93.5 (91.5-95.5) | 0.6395 |
| LBC≥ASCUS and/or CISCER(+) | 0.684 (0.664-0.703) | 100 (100-100) | 36.7 (32.8-40.6) | 11.5 (8.5-14.6) | 100 (100-100) | 0.003 |
| HPV33/35(+) and/or LBC≥ASCUS | 0.622 (0.572-0.673) | 87.8 (78.6-96.9) | 36.7 (32.8-40.6) | 10.3 (7.4-13.2) | 97.3 (95.2-99.4) | 0.1863 |
| HPV33/35(+) and/or CISCER(+) | 0.862 (0.817-0.908) | 89.8 (81.3-98.3) | 82.7 (79.6-85.7) | 29.9 (22.5-37.3) | 99 (98.1-99.9) | <0.001 |
| HPV33/35(+) and/or LBC≥ASCUS and/or CISCER(+) | 0.671 (0.652-0.69) | 100 (100-100) | 34.2 (30.4-38) | 11.1 (8.2-14.1) | 100 (100-100) | <0.001 |

DeLong test: with LBC≥ASCUS as the comparator.

95% CI: 95% confidence interval; ASCUS: liquid-based cytology results were atypical squamous cells of undetermined significance or worse; AUC: area under the curve of the receiver operating characteristic; CISCER(+): ΔCt *PAX1* ≤ 6.6 or ΔCt *JAM3*≤10.0; HPV33/35: HPV33(+) or HPV35(+); LBC≥ NPV, negative predictive value; PPV: positive predictive value.
