# Supplementary material for: The triage role of cytological DNA methylation in women with non-16/18, specifically genotyping high-risk HPV infection
Source: Br J Cancer. 2025 Apr 10;132(11):1064–71. doi: 10.1038/s41416-025-03005-5 (PMC12119919; doi:10.1038/s41416-025-03005-5)
Supplement: Supplementary file 2 — Table S2. Absolute CIN2+ and CIN3+ risks for single and combined triage tests. [file 41416_2025_3005_MOESM2_ESM.docx]

**Supplement Table S2. Absolute CIN2+ and CIN3+ risks for single and combined triage tests**

|  | N | Colposcopy referral (%) | CIN2+ (n^a^) | Absolute CIN2+ risk(95%CI) | Referrals needed to detect CIN2+ | CIN3+(n^b^) | Absolute CIN3+ risk(95%CI) | Referrals needed to detect CIN3+ |
| --- | --- | --- | --- | --- | --- | --- | --- | --- |
| ALL | 643 |  | 113 | 17.6% (14.7%-20.7%) | 5.7 | 49 | 7.6% (5.7%-9.9%) | 13.1 |
| **Single triage strategies** |  |  |  |  |  |  |  |  |
| LBC - | 245 | 38.1% | 24 | 9.8% (6.4%-14.2%) | 10.2 | 10 | 4.1% (2.0%-7.4%) | 24.5 |
| LBC+ | 398 | 61.9% | 89 | 22.4% (18.4%-26.8%) | 4.5 | 39 | 9.8% (7.1%-13.2%) | 10.2 |
| CISCER- | 531 | 82.6% | 27 | 5.1% (3.4%-7.3%) | 19.7 | 5 | 0.9% (0.3%-2.2%) | 106.2 |
| CISCER+ | 112 | 17.4% | 86 | 76.8% (67.9%-84.2%) | 1.3 | 44 | 39.3% (30.2%-49.0%) | 2.5 |
| HPV33/35- | 586 | 91.1% | 88 | 15.0% (12.2%-18.2%) | 6.7 | 38 | 6.5% (4.6%-8.8%) | 15.4 |
| HPV33/35+ | 57 | 8.9% | 25 | 43.9% (30.7%-57.6%) | 2.3 | 11 | 19.3% (10.0%-31.9%) | 5.2 |
| **Combined triage strategies** |  |  |  |  |  |  |  |  |
| LBC- and CISCER- | 218 | 33.9% | 7 | 3.2% (1.3%-6.5%) | 31.1 | 0 | 0.0% (0.0%-1.7%) | NA |
| LBC- and CISCER+ | 27 | 4.2% | 17 | 63.0% (42.4%-80.6%) | 1.6 | 10 | 37.0% (19.4%-57.6%) | 2.7 |
| LBC+ and CISCER- | 313 | 48.7% | 30 | 9.6% (6.6%-13.4%) | 10.4 | 5 | 1.6% (0.5%-3.7%) | 62.6 |
| LBC+ and CISCER+ | 85 | 13.2% | 69 | 81.2% (71.2%-88.8%) | 1.2 | 34 | 40.0% (29.5%-51.2%) | 2.5 |
| LBC- and HPV33/35+ | 21 | 3.3% | 9 | 42.9% (21.8%-66.0%) | 2.3 | 4 | 19.0% (5.4%-41.9%) | 5.2 |
| LBC- and HPV33/35- | 224 | 34.8% | 15 | 6.7% (3.8%-10.8%) | 14.9 | 6 | 2.7% (1.0%-5.7%) | 37.3 |
| LBC+ and HPV33/35+ | 36 | 5.6% | 16 | 44.4% (27.9%-61.9%) | 2.2 | 7 | 19.4% (8.2%-36.0%) | 5.1 |
| LBC+ and HPV33/35- | 362 | 56.3% | 73 | 20.2% (16.2%-24.7%) | 5 | 32 | 8.8% (6.1%-12.2%) | 11.3 |
| HPV33/35- and CISCER- | 496 | 77.1% | 22 | 4.4% (2.8%-6.6%) | 22.5 | 5 | 1.0% (0.3%-2.3%) | 99.2 |
| HPV33/35- and CISCER+ | 90 | 14.0% | 66 | 73.3% (63.0%-82.1%) | 1.4 | 33 | 36.7% (26.8%-47.5%) | 2.7 |
| HPV33/35+ and CISCER- | 35 | 5.4% | 5 | 14.3% (4.8%-30.3%) | 7 | 0 | 0.0% (0.0%-10.0%) | NA |
| HPV33/35+ and CISCER+ | 22 | 3.4% | 20 | 90.9% (70.8%-98.9%) | 1.1 | 11 | 50.0% (28.2%-71.8%) | 2 |
| LBC- and HPV33/35- and CISCER- | 203 | 31.6% | 4 | 2.0% (0.5%-5.0%) | 50.8 | 0 | 0.0% (0.0%-1.8%) | NA |
| LBC- and HPV33/35- and CISCER+ | 21 | 3.3% | 11 | 52.4% (29.8%-74.3%) | 1.9 | 2 | 9.5% (1.2%-30.4%) | 10.5 |
| LBC- and HPV33/35+ and CISCER- | 15 | 2.3% | 3 | 20.0% (4.3%-48.1%) | 5 | 0 | 0.0% (0.0%-21.8%) | NA |
| LBC- and HPV33/35+ and CISCER+ | 6 | 0.9% | 6 | 100.0% (54.1%-100.0%) | 1 | 4 | 66.7% (22.3%-95.7%) | 1.5 |
| LBC+ and HPV33/35- and CISCER- | 293 | 45.6% | 18 | 6.1% (3.7%-9.5%) | 16.3 | 5 | 1.7% (0.6%-3.9%) | 58.6 |
| LBC+ and HPV33/35- and CISCER+ | 69 | 10.7% | 55 | 79.7% (68.3%-88.4%) | 1.3 | 27 | 39.1% (27.6%-51.6%) | 2.6 |
| LBC+ and HPV33/35+ and CISCER- | 20 | 3.1% | 2 | 10.0% (1.2%-31.7%) | 10 | 0 | 0.0% (0.0%-16.8%) | NA |
| LBC+ and HPV33/35+ and CISCER+ | 16 | 2.5% | 14 | 87.5% (61.7%-98.4%) | 1.1 | 7 | 43.8% (19.8%-70.1%) | 2.3 |

N: number of total ; na: number of CIN2+ detected; nb: number of CIN3+ detected.

95% CI: 95% confidence interval; CISCER-: ΔCt *PAX1* > 6.6 and ΔCt *JAM3*>10.0; CISCER+: ΔCt *PAX1* ≤ 6.6 or ΔCt *JAM3*≤10.0; HPV33/35+: HPV33(+) or HPV35(+); HPV33/35-: HPV31, 39, 45, 51, 52, 56, 58, 59, 66, and 68, with positivity for one or more types; LBC-:liquid-based cytology results are classified as no intraepithelial lesions or malignancy; LBC+:liquid-based cytology results are classified as atypical squamous cells of undetermined significance or worse; OR: odds ratio.
